# The Psycho-cardiac Coupling, Myocardial Remodeling, and Neuroendocrine Factor Levels: The Psychosomatics of Major Depressive Disorder

**DOI:** 10.7759/cureus.2464

**Published:** 2018-04-11

**Authors:** Javeria N Syeda, Ian H Rutkofsky, Adnan S Muhammad, Tarig H Balla Abdalla, Zahid Saghir

**Affiliations:** 1 Department of Research, California Institute of Behavioral Neurosciences & Psychology, Fairfield, CA, USA

**Keywords:** heart depression, cardiac depression, depression myocardium, depression epicardium, mi depression, depression endocardium

## Abstract

The association of major depressive disorder (MDD) with myocardial infarction (MI) and vice versa is not unknown. Depression, along with many other systemic factors like atherosclerosis, obesity, diabetes and vascular dysfunction, contributes to the development of adverse cardiac events in the future and, has always been a topic of interest in the fields of cardiology and psychosomatics. We wrote this review article to elaborate this relationship in detail. This article suggests that the individuals with type D personality who already had cardiovascular disease had undergone more serious myocardial damage. In addition, we elucidated the effects of depression on sympathetic activity and remodeling of myocardium after MI. The alterations in the neuroendocrine factors, which included the changes in levels of Serotonin (5-HT), Norepinephrine and Corticosterone, also geared towards the changes associated with depression-induced myocardial injury. However, we need more studies in the near future to further dig into this association process. Therefore, we recommend more research to explore the relationship of psychological factors and adverse cardiac outcomes.

## Introduction and background

“At the root of this dilemma is the way we view mental health in this country. Whether an illness affects your heart, your leg or your brain, it’s still an illness, and there should be no distinction.”- Michelle Obama.

A 45-year-old female with the past medical history of systemic lupus erythematosus (SLE) and hypertension (HTN) came to the emergency department with the complaints of chest pain and diaphoresis for the past hour. She was diagnosed with SLE 10 years ago and was taking multiple drugs for controlling her flare-ups. She was also undergoing psychotherapy sessions once a week due to the newly made diagnosis of major depressive disorder (MDD). On admission, her vitals were a blood pressure of 140/80 mmHg, a pulse of 95/min, and respiratory rate of 20/min. Her initial labs showed troponin levels as undetectable that later turned out to be positive. Electrocardiography performed showed ST elevation in leads V1 to V4. Coronary angiography performed showed occlusion of the proximal left anterior descending artery (LAD). An initial diagnosis of myocardial infarction (MI) was made and the patient was admitted to the telemetry unit. That is how the patients with psychosomatic problems usually present.

In 2015, there were 216 million people affected by MDD which is around 3% of the world's population [1]. The people affected vary in percentage from 7% in Japan to 21% in France [2]. The rates are different in the developing world (11%) as compared to that in the developed world (15%) [2].

The term “psychosomatic illness” is defined as any physical condition which involves organic or functional damage affected by psychological factors in the process of its inception or development [3]. This definition is in accordance with 'psychological factors affecting medical conditions' (code 316), which appears in the Diagnostics and Statistical Manual of Mental disorders, ed. 4, text revision (DSM-IV-TR) published by the American Psychiatric Association [3]. Likewise, behavioral medicine can be defined as an interdisciplinary field concerned with the development and assimilation of biomedical and behavioral knowledge related to health and disease. It also applies this knowledge to the prevention, diagnosis, treatment, and care. The scope of behavioral medicine incorporates bio-behavioral mechanisms (i.e., the synergy of biomedical processes with psychological, social, societal, cultural, and environmental processes), for the clinical diagnosis and intervention, along with public health [4].

MDD, which is commonly known as depression, is defined by the presence of five (or more) of the following symptoms that are present for a period of two weeks; one of these symptoms is either depressed mood or an inhibition of interest:

• Depressed mood most of the day.

• Markedly inhibited interest in all, or almost all activities.

• Marked unintended weight loss or weight gain.

• Changes in sleep (decreased or increased the need for sleep) nearly every day.

• Psychomotor changes nearly every day.

• Fatigue or tiredness nearly every day.

• Feelings of guilt or worthlessness nearly every day.

• Decreased ability to think or concentrate nearly every day.

• Recurrent thoughts of death, or recurrent suicidal ideation, or a suicide attempt [5]. On average, MDD is the reason for 11% of the years lived with a disability, and up to 15% of the patients with recurrent MDD end up committing suicide [6].

There is a general concept of stress being a risk factor for the development of heart attack, however, little is known about the association of major depression with MI. Development of depression in patients with the heart attack is a common occurrence. However, if all of the criteria for MDD or some of them are met and if this association is long lived or short lived is not yet known. The association of MDD with a heart attack can be categorized into three factors. Either of them can be associated with the other in a positive or negative way or not be associated with each other at all. “What are the major symptoms encountered in patients who do develop depression after heart events?”, “Are there any ways by which we can prevent this from happening?”, and “What is the percentage of suicide in such patients?” are all such questions which still need to be answered.

We will discuss the association of MI with depression in great detail. We will mention the research on the patients with type D personality who developed acute coronary syndrome (ACS) later on with the statistically significant values in their troponin and myoglobin levels [7]. Also, the independent relationship of depression with MI in accordance with the activities in their sympathetic and vagal systems will be highlighted [8]. The changes induced by chronic unpredictable mild stress (CUMS) on the neuroendocrine factors will also be included which will highly correlate the relationship depression has with myocardial apoptosis [9]. Lastly, we will focus on the psychological effects on the patients after having undergone MI and how to prevent them [10-11].

## Review

We wrote this review article on the subject of the association of major depression with the heart walls. Reviews on depression and heart were conducted in PubMed, MEDLINE and PubMed Central, PsycINFO, Cochrane library, and various newspapers. Cross-checking of references led to the additional articles. The decision to include or exclude reviews and data extraction was made after deciding on the inclusion and exclusion criteria and any disagreements were settled by discussion. Articles on patients with depression and with bodily symptoms (cardiac symptoms) were thoroughly searched and later the articles focusing mostly on the association between depression and heart were included. The reviews which had a high possibility of bias and the studies with confusing data were excluded. Moreover, the in vitro studies were also excluded. The keywords for the data search included: Heart depression (which gave 25,072 articles), cardiac depression (which gave 29,121 articles), MI and depression (which gave 5893 articles), depression myocardium (which gave 3861 articles), depression epicardium (which gave 123 articles), depression endocardium (which gave 71 articles). A total of 64,141 articles initially showed up and after excluding the repetition and the irrelevant articles, we finally included only 30 relevant studies which focused on the major depression and the myocardium.

The psychosomatics of major depression and myocardium

MI is defined as the death of the myocardium because of prolonged ischemia [12]. The process of necrosis occurs due to the imbalance between oxygen demand and supply [13]. This can be further explained in a way that whenever there is any discrepancy between the oxygen supply, i.e., hypoxemia, anemia, hypotension, etc. and in the demand, i.e., tachycardia, tachyarrhythmias, hypertension, etc., then there is damage to the myocardium [14]. MI is caused by an acute coronary event followed by plaque rupture [14]. There is ample evidence suggesting that there is a strong linkage between type D personality and development of ischemic heart diseases and MI. Type D personality is the term which is defined as a propensity of having negative sentiments, i.e., sadness, constant fret, and grumpiness. D denotes for being “distressed” [15-16]. A study was conducted to investigate the relationship between type D (Depressed) personality and cardiac biomarkers in patients with the acute coronary syndrome. A total of 215 patients with ACS were given a survey which included the measure of type D personality. Their samples of blood were taken within three days of the ischemic event which included lipid profiles and cardiac enzymes. The conclusion of this cross-sectional study was that the patients who already had cardiovascular disease and type D personality were associated with more damage to the myocardium. It could be explained by the presence of ST elevation (R^2^ = 0.07) along with higher troponin (R^2^ = 0.05) and myoglobin levels (R^2^ = 0.07) [7].

**Figure 1:**
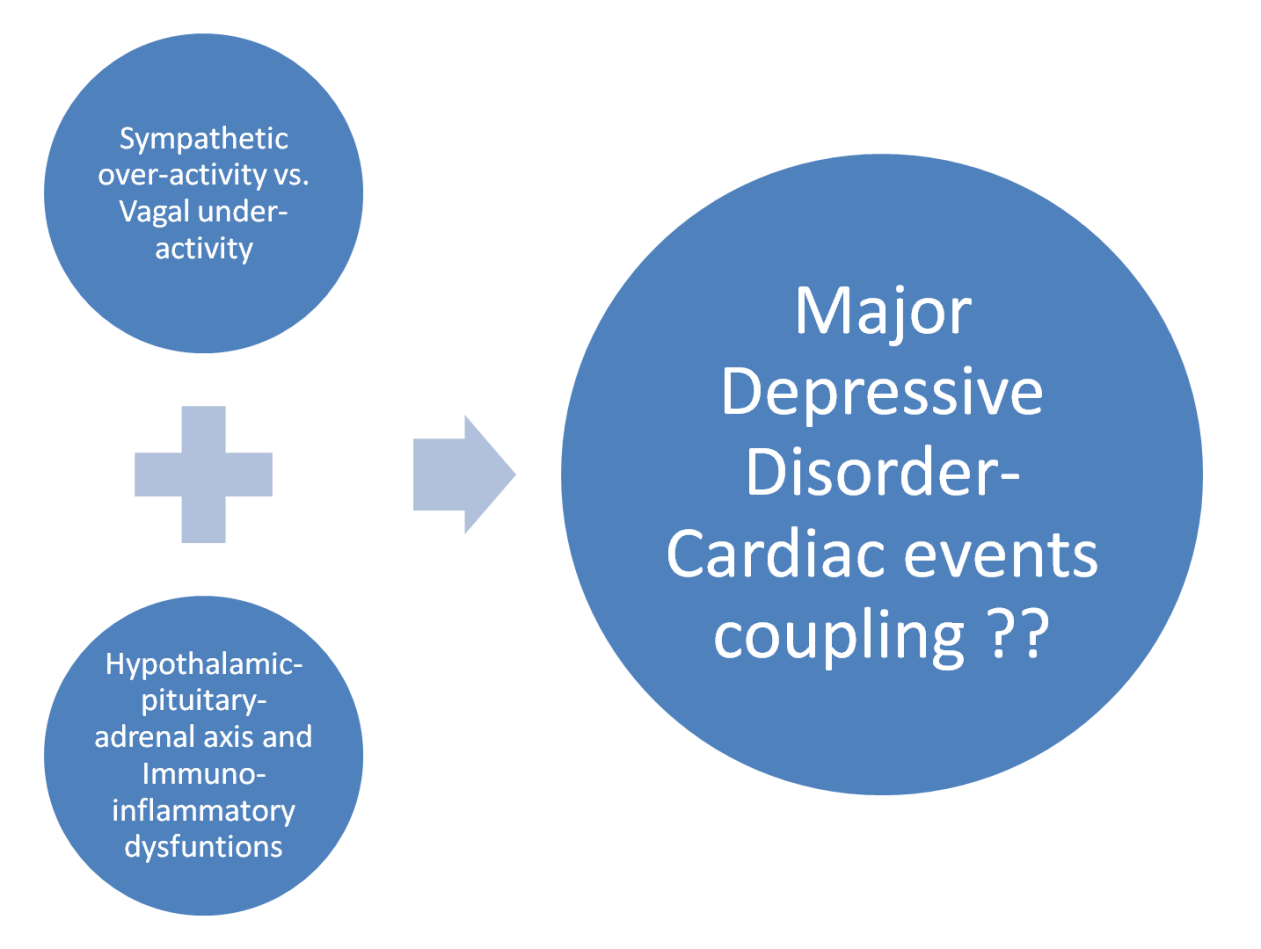
The processes involved in the psycho-cardiac coupling.

MDD increases both the risks of ischemic or coronary heart disease (CHD) and the resultant morbidity and mortality. The mechanism of this linkage takes into account all the systemic factors including vascular dysfunction, atherosclerosis, obesity, and diabetes, which altogether increase the risk of CHD. Regardless, there is an ample evidence already proved in the experiments that say that the myocardium is directly altered in depression, independently of these factors. It is postulated that MDD is involved in impairing the heart’s intrinsic defense mechanisms. There are certain processes that are incriminated in this causality which might be the target for future medical interventions. They include sympathetic over-activity vs. vagal under-activity, along with hypothalamic-pituitary-adrenal (HPA) axis and immuno-inflammatory dysfunctions, which is further elaborated in the figure below (Figure 1). However, data regarding their involvement is scarce and whether or not, the cardiac sequelae correlated with MDD would be halted after controlling these mediators is yet to be known [8].

There was one more study done to elucidate the effect of depression on sympathetic activity and remodeling of myocardium after MI. Wild-type rats were taken and were divided into four groups: Sham group (sham), MI group, depression group (D), and MI plus depression group (MI+D). When the groups were compared with controls, the MI +D group showed decreased weight gain and depression-like symptoms. When the sympathetic activity was observed, it displayed an increase in the plasma levels of epinephrine and norepinephrine along with higher expression of myocardial tyrosine hydroxylase in the MI+D group than control group (p < 0.05 for all the results). When the function and morphology of the heart were analyzed, it showed decreased fractional shortening along with increased left ventricular dimensions, thinning of the myocardial wall and decreased collagen repair in the MI+D group compared with the MI group (p < 0.05 for all) [17].

**Figure 2:**
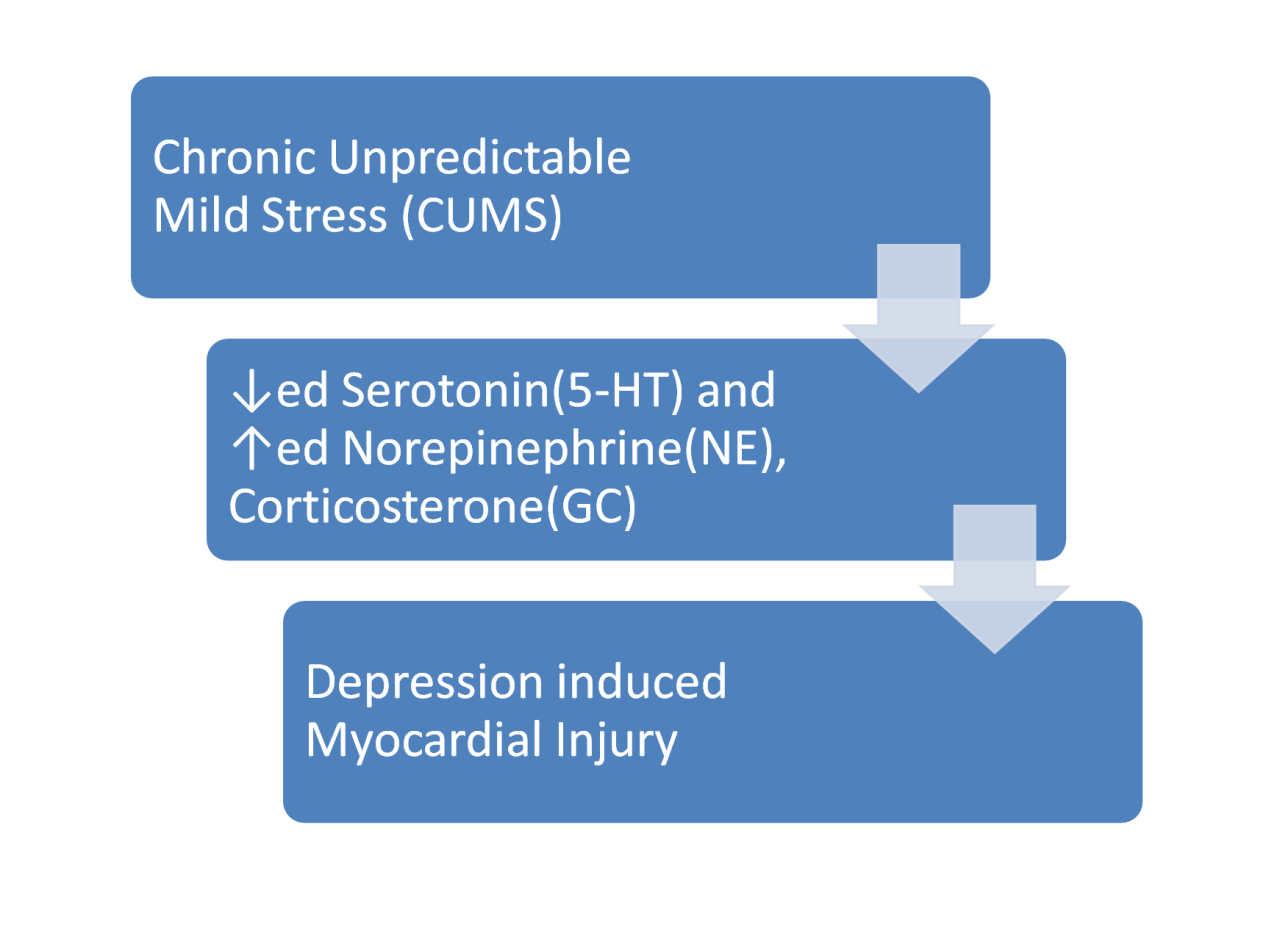
The relationship of neuroendocrine factors in CUMS rat models. CUMS: Chronic unpredictable mild stress.

Another study was performed to ascertain the effects of depression induced by CUMS on myocardial injury and to explain the mechanism of depression. Once again, rats were used as a model. The procedure of CUMS went on for about eight weeks. When four weeks went by, the rats, who were treated, showed a reduced preference for sucrose and revised scores in an open field test, body weight and the amount of 5-HT in the brain in comparison with the controls. Such changes pointed to depression-like changes. Moreover, the alterations that were noted after six weeks of CUMS were increased significantly after eight weeks of CUMS which included enhanced myocardial apoptosis. The plasma levels of neuroendocrine factors, i.e. serotonin (5-HT), norepinephrine (NE) and epinephrine (E), were measured along with plasma corticosterone (GC) in controls and CUMS rats. The results concluded that 5-HT had decreased whereas NE, E, and GC had increased in the CUMS rats which proved that these markers could be associated with depression-induced myocardial injury. In addition to that, an orthogonal design was made which showed the effects of 5-HT, NE, and GC on the myocardial survival. According to that design, 5-HT had a more integral part in cell survival than NE and GC. The final conclusion in accordance with the myocardial injury in relation to depression was proved by the decreased level of 5-HT along with the increased levels of NE and GC, which is shown and elucidated in Figure 2 [9].

The linkage of depression and MI could also be explained by the increased expression of coagulation factors and decreased fibrinolysis in a depressed individual. A study was conducted to determine an association between depression/anxiety and coagulation factors with fibrinolysis in patients who underwent MI in the last three months. A total of 148 patients were enrolled in the study among which anxiety and depression were evaluated by the hospital anxiety and depression scale (HADS) shortly and after three months in patients who had MI. At their second round of evaluation, some additional factors were also observed which included the plasma levels of fibrinogen, factor VII, factor VIII, von Willebrand factor, prothrombin-fragment 1 and 2, tissue-plasminogen activator, plasminogen activator inhibitor-1, D- dimer and homocysteine. The results concluded that increased levels of anxiety and depression were found in 32% of patients after three months of MI. Coagulation and fibrinolysis markers were not correlated with HADS depression and anxiety scores according to multiple regression analyses. It was also concluded that age, gender, body mass index, and smoking status were significantly associated with hemostasis factors. A higher age was correlated with a higher propensity of coagulability but lower anxiety levels [18].

In the previous paragraphs, we emphasized that depression could lead to adverse cardiac outcomes. Likewise, cardiac events could also lead to adverse psychological outcomes in the long run. The processes behind this linkage are still not known but it has been hypothesized that there might be an increase in pro-apoptotic pathways in the myocardium and hippocampus in MDD [10]. There is some evidence that shows that antidepressants can protect the heart if given to the patients who have suffered MI which was associated with depression. A study was conducted to determine the mechanism of escitalopram at a molecular level on myocardial apoptosis and Bax, Bcl-2 expression in rats of depression during myocardial ischemia/reperfusion (I/R). A total of eight rats were taken and were divided into three groups: D group (depression), D I/R group (depression with myocardial I/R) and escitalopram + D I/R group. All the three groups were given the same amount of CUMS and were separated for 21 days. At the same time, the last group was given escitalopram by gavage (10 mg/kg/day) and the rats in the myocardial I/R group had their left anterior descending arteries ligated. The final results were as follows: The rats in escitalopram + D I/R group showed increased movements and sucrose consumption as compared to the D and D I/R group (p < 0.01). Also, the infarct size of the myocardium in the escitalopram + D I/R group was significantly decreased as compared to the D I/R group (p < 0.01). And there was downregulation of Bax and Bcl-2 ratio in the escitalopram + D I/R group as compared to the D I/R group (p < 0.01) [11].

## Conclusions

Major depression has an effect on sympathetic activity and leads to cardiac remodeling. Heart's intrinsic defence mechanism is also altered because of major depression. Animal studies with rats suggest that depression can increase the norepinephrine, epinephrine, and corticosterone levels while reducing the serotonin levels. Moreover, the increase in the pro-apoptotic pathways in the myocardium and hippocampus in the major depressive patients is a possibility. Yet it is inconclusive because some studies are animal based. We surely need more human-based observations to strengthen the evidence quality in the coming future. With all the above-mentioned evidence, why do all depressed patients not develop myocardial damage remains inconclusive. We will have to wait for more data in the coming decades to understand more about this association.
